# Morphological features of the greater occipital nerve and its possible importance for interventional procedures

**DOI:** 10.1111/joa.13959

**Published:** 2023-09-30

**Authors:** Latif Saglam, Ozcan Gayretli, Osman Coskun, Aysin Kale

**Affiliations:** ^1^ Department of Anatomy, Istanbul Faculty of Medicine Istanbul University Istanbul Turkey

**Keywords:** block, decompression surgery, greater occipital nerve, headache, occipital artery

## Abstract

Being one of the most prevalent neurological symptoms, headaches are burdensome and costly. Blocks and decompression surgeries of the greater occipital nerve (GON) have been frequently used for migraine, cervicogenic headache, and occipital neuralgia which are classified under headache by International Headache Society. Knowledge of complex anatomy of GON is crucial for its decompression surgery and block. This study was performed to elucidate anatomical features of this nerve in detail. Forty‐one cadavers were dissected bilaterally. According to its morphological features, GON was classified into four main types that included 18 subtypes. Moreover, potential compression points of the nerve were defined. The number of branches of the GON up to semispinalis capitis muscle and the number of its branches that were sent to this muscle were recorded. The most common variant was that the GON pierced the aponeurosis of the trapezius muscle, curved around the lower edge of the obliquus capitis inferior muscle, and was loosely attached to the obliquus capitis inferior muscle (Type 2; 61 sides, 74.4%). In the subtypes, the most common form was Type 2‐A (44 sides, 53.6%), in which the GON pierced the aponeurosis of each of the trapezius muscle and fibers of semispinalis muscle at one point and there was a single crossing of the GON and occipital artery. Six potential compression points of the GON were detected. The first point was where the nerve crossed the lower border of the obliquus capitis inferior muscle. The second and third points were at its piercing of the semispinalis capitis muscle and the muscle fibers/aponeurosis of the trapezius, respectively. Fourth, fifth, and sixth compression points of GON were located where the GON and occipital artery crossed each other for the first, second, and third times, respectively. On 69 sides, 1–4 branches of the GON up to the semispinalis capitis muscle were observed (median = 1), while 1–4 branches of GON were sent to the semispinalis capitis muscle on 67 sides (median = 1). The novel anatomical findings described in this study may play a significant role in increasing the success rate of invasive interventions related with the GON.

## Graphical abstract

The largest report of detailed morphological classification of the greater occipital nerve has been studied.

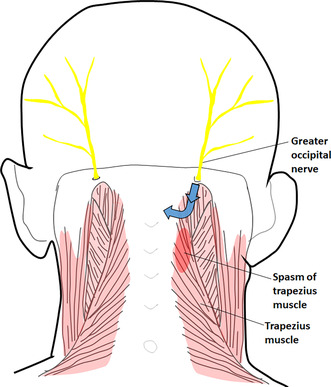

## 1 INTRODUCTION

Headache is one of the most common types of pain and the most common cause of admission to outpatient clinics. It seriously decreases the quality of life and can accompany many systemic diseases as well as neurological disorders (Özge et al., 2021). The prevalence of headache varies across the continents: reported to be about 20% in Africa, and approximately 50% in other continents (Stovner et al., 2007). It is also more prevalent in females than males worldwide (Saylor & Steiner, 2018; Stovner et al., 2007).

Greater occipital nerve block (GONB) is an effective treatment in migraine (Allen et al., 2018; Gawel & Rothbart, 1992; Malekian et al., 2021; Tang et al., 2017; Zhang et al., 2018), cervicogenic headache (Anthony, 2000; Caponnetto et al., 2021; Inan et al., 2001; Lauretti et al., 2015), and occipital neuralgia (Anthony, 1992; Ashkenazi et al., 2007; Ashkenazi & Levin, 2004; Newman & Levin, 2008), which are among the causes of headache. The greater occipital nerve (GON) is the medial branch of dorsal ramus of second cervical spinal nerve (Standring, 2021). It curves around lower edge of the obliquus capitis inferior muscle (OCI), deep to semispinalis capitis muscle (SS), and supplies SS (Standring, 2021). GON pierces SS, afterward, it passes through an aponeurotic sling that is extended between trapezius muscle (TM) and sternocleidomastoid muscle (SCM) close to their occipital insertions (Standring, 2021). It is together with occipital artery (OA) during its course on the scalp (Standring, 2021). Various anatomical landmarks are determined for applying GONB (Austad, 2004; Caputi & Firetto, 1997; Kwon et al., 2018; Loukas et al., 2006; Mosser et al., 2004; Young, 2010). Success of GONB may vary depending on the techniques used, concerning the landmarks (Nair et al., 2018). On the other hand, in some cases, GONB does not alleviate these symptoms (Dilli et al., 2015; Eskilsson et al., 2021; Kashipazha et al., 2014), possibly due to anatomical variations and/or differences in the topographical location of GON (Ducic et al., 2009; Huanmanop et al., 2021; Junewicz et al., 2013; Natsis et al., 2006; Won et al., 2018). Although it is considered to be safe, repeated corticosteroid injections may cause complications such as Cushing's syndrome (Lavin & Workman, 2001) or neck muscle weakness (Ducic et al., 2009).

Similarly, decompression surgery for GON can be used therapeutically in migraine, cervicogenic headache, and occipital neuralgia (Ducic et al., 2009; Guyuron et al., 2005; Magnússon et al., 1996). The aim of the decompression surgery of GON is to release potential compression points of GON and liberate the nerve (Bovim et al., 1992; Muehlberger, 2018). Knowing the anatomy and potential entrapment points of GON in detail is essential to achieve a successful decompression surgery (Baldelli et al., 2020).

Several studies related with the morphological features of GON have been performed previously. Nevertheless, the number of branches of GON and detailed information about decussation of GON and OA have not been reported yet. Moreover, few studies have focused on the topographical location of GON. This study aims to elucidate morphological features of GON so that its anatomical properties are better defined and the data obtained may give support to the related clinical procedures.

## 2 METHODS

Occipital region of 27 males and 14 females, totally 41 cadavers, aged between 35 and 88 years, were dissected bilaterally by two experienced anatomists (LS and OG) at the Department of Anatomy, Istanbul Faculty of Medicine. None of the cadaveric specimens revealed any evidence of gross pathology and traumatic lesions of the occipital region including posterior part of the scalp. The present study was approved by the Clinical Research Ethical Committee of Istanbul Faculty of Medicine (IRB—institutional review board; number: 2021/370).

Specimens were organized in prone position. Scalp covering posterior part of cranium was shaved in each cadaver. A vertical incision extending from a point located approximately 5 cm above the external occipital protuberance (EOP) to spinous process of the prominent vertebra was performed and this incision passed through the EOP. Afterward, two transverse incisions of approximately 10‐cm length were accurately carried out from the upper and lower borders of the previous vertical incision. Dissecting the skin, subcutaneous tissue, and fascias, TM, splenius capitis muscle (SC), and SCM were exposed. Subsequently, these muscles were dissected and deviated from medial to lateral carefully, making SS visible. Afterward deviating SS from medial to lateral, the suboccipital muscles which were deeply located to SS were exposed. GON was identified and particularly carefully dissected and sketched, with special attention being paid to the course of it. The dissection was carried out proximally to reach its emergence under OCI. Along with the course of GON, branches of it were revealed clearly. The relationship between OA and GON was also explored throughout the whole dissection.

Each GON was evaluated morphologically and a classification was formed according to the data obtained.

### 2.1 Classification

*Type 1*: GON pierces fibers of TM, curves around lower edge of OCI, and it is attached loosely to this muscle.

**FIGURE 1:**
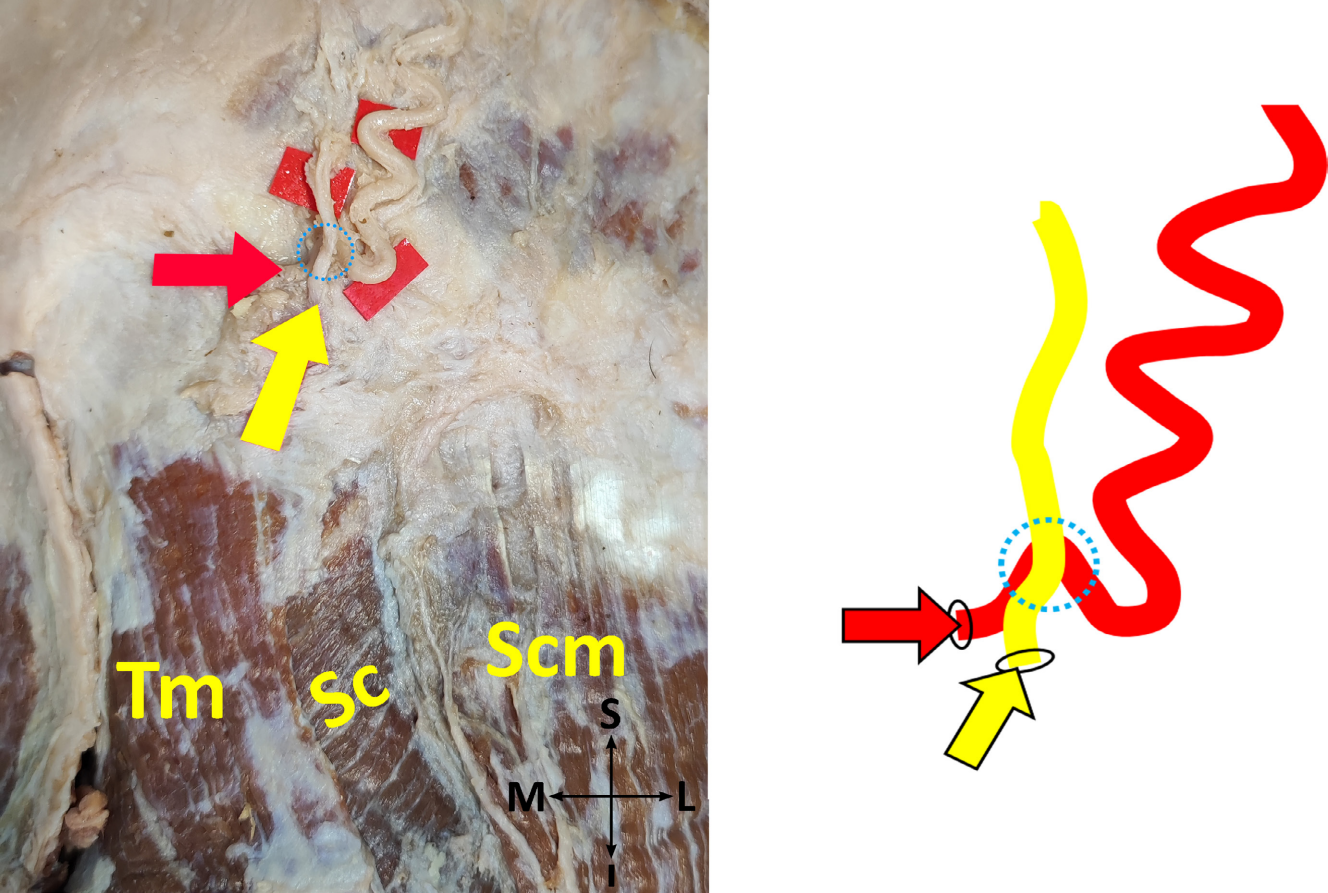
A single cross of the greater occipital nerve and occipital artery on the cadaver (on the left side), and its schematic illustration (on the right side) (posterior view, right). I, inferior; L, lateral; M, medial; S, superior; SC, splenius capitis muscle; SCM, sternocleidomastoid muscle; TM, trapezius muscle; Yellow arrow, the point where the greater occipital nerve exits. Red arrow, the point where the occipital artery exits. Dotted blue circle, the point where the greater occipital nerve and occipital artery cross each other.

*Subtype 1‐A*: GON pierces fibers of each TM and SS at one point, a single cross of GON and OA exists (Figure 1).

**FIGURE 2:**
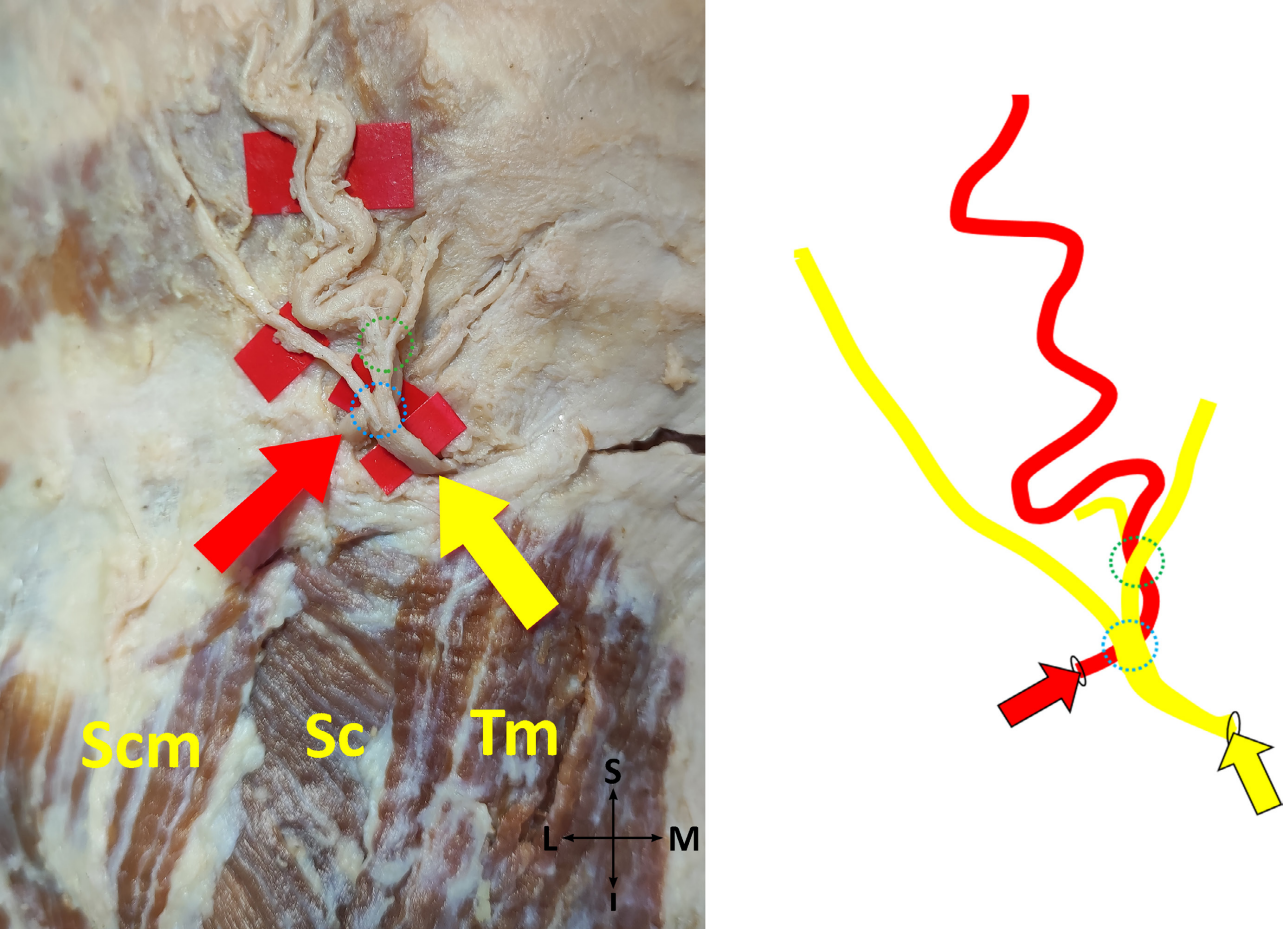
Two crosses of the greater occipital nerve and occipital artery on the cadaver (on the left side), and its schematic illustration (on the right side) (posterior view, left). I, inferior; L, lateral; M, medial; S, superior; SC, splenius capitis muscle; SCM, sternocleidomastoid muscle; TM, trapezius muscle; Yellow arrow, the point where the greater occipital nerve exits. Red arrow, the point where the occipital artery exits. Dotted blue circle, first point where the greater occipital nerve and occipital artery cross each other. Dotted green circle, second point where the greater occipital nerve and occipital artery cross each other.

*Subtype 1‐B*: GON pierces fibers of each TM and SS at one point, two crosses of GON and OA exist (Figure 2).

**FIGURE 3:**
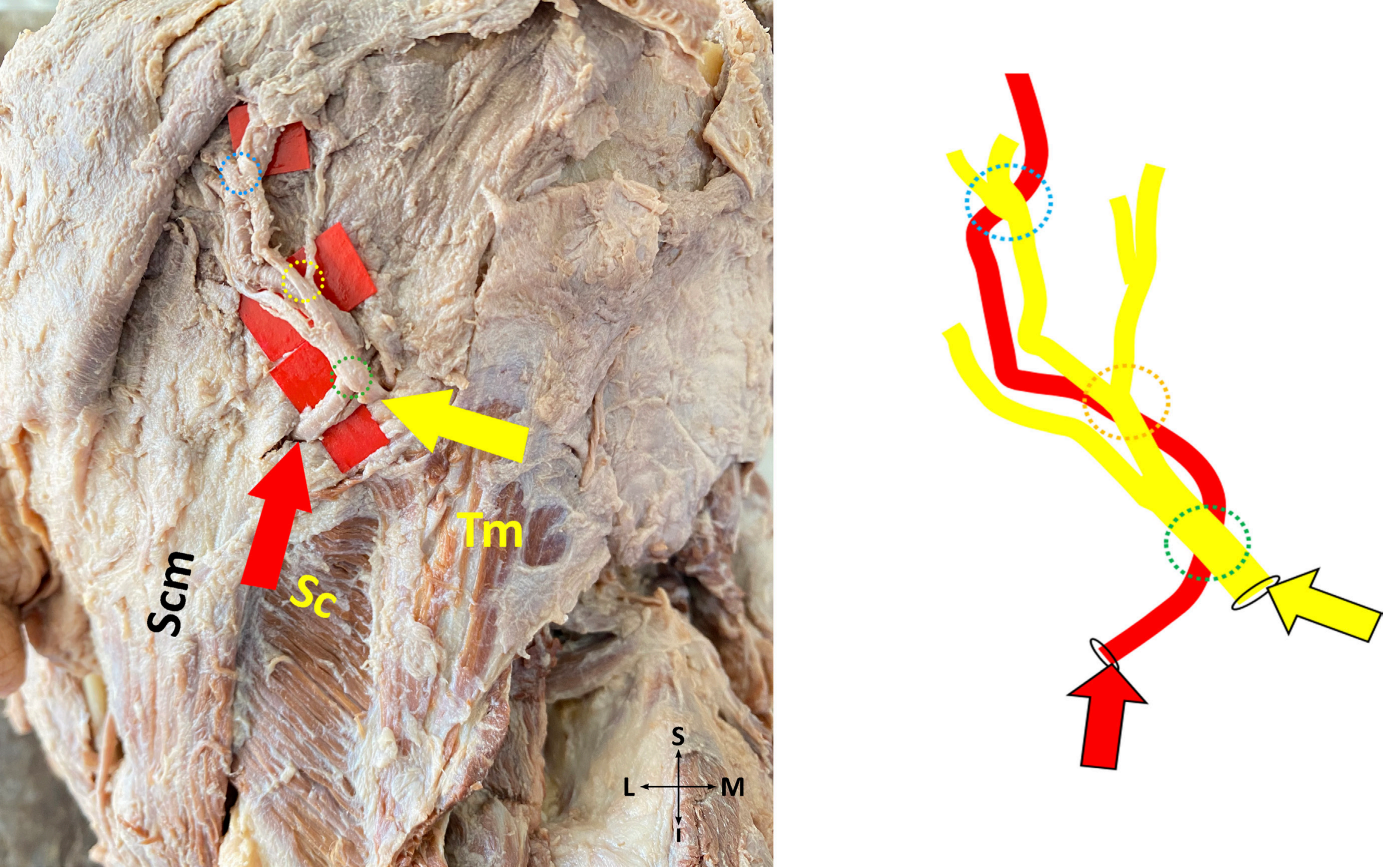
Three crosses of greater occipital nerve and occipital artery on the cadaver (on the left side), and its schematic illustration (on the right side) (posterior view, left). I, inferior; L, lateral; M, medial; S, superior; SC, splenius capitis muscle; SCM, sternocleidomastoid muscle; TM, trapezius muscle; Yellow arrow, the point where the greater occipital nerve exits. Red arrow, the point where the occipital artery exits. Dotted green circle, first point where the greater occipital nerve and occipital artery cross each other. Dotted yellow circle, second point where the greater occipital nerve and occipital artery cross each other. Dotted blue circle, third point where the greater occipital nerve and occipital artery cross each other.

*Subtype 1‐C*: GON pierces fibers of each TM and SS at one point, three crosses of GON and OA exist (Figure 3).

**FIGURE 4:**
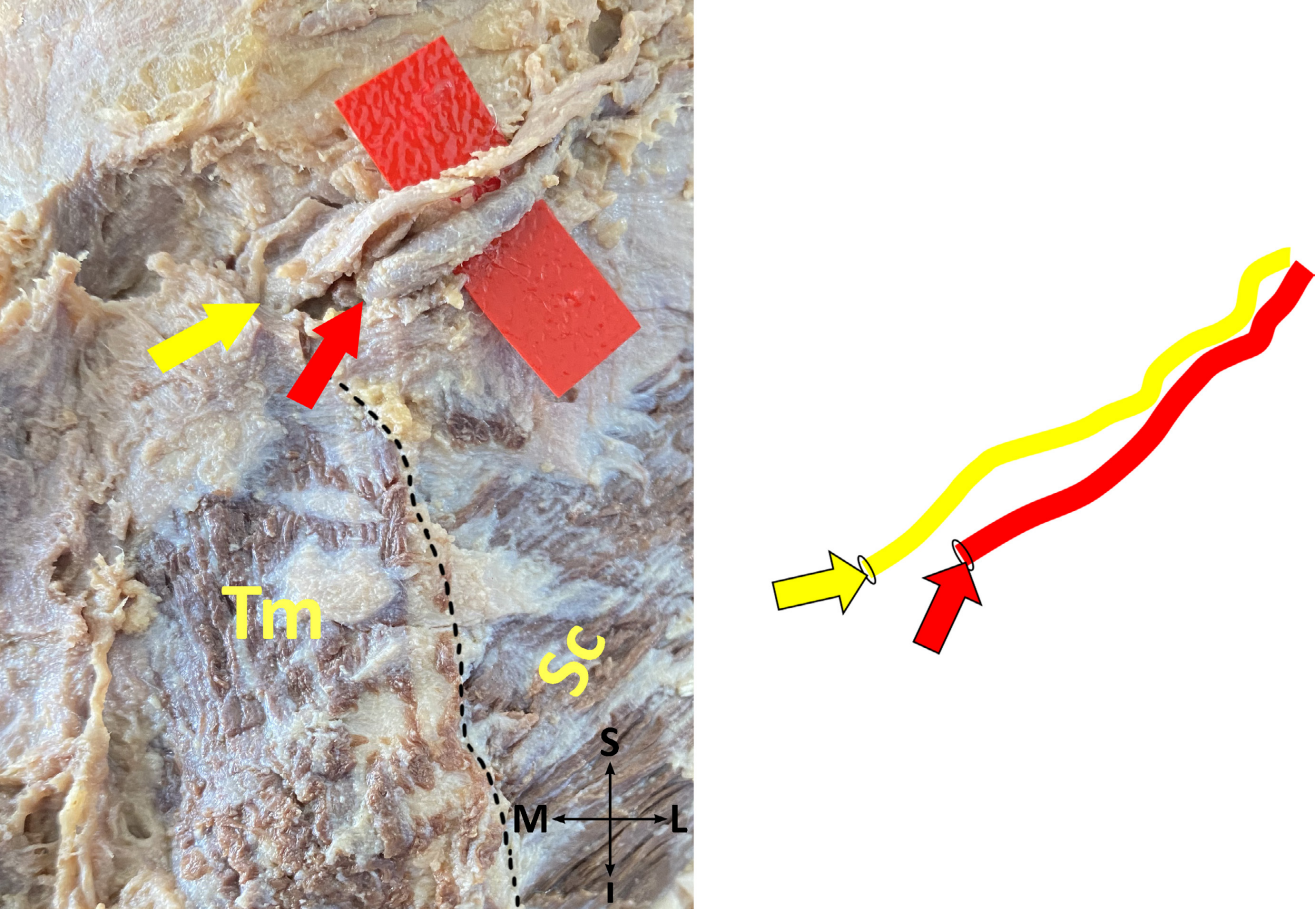
No cross of the greater occipital nerve and occipital artery on the cadaver (on the left side), and its schematic illustration (on the right side) (posterior view, right). I, inferior; L, lateral; M, medial; S, superior; SC, splenius capitis muscle; TM, trapezius muscle; Yellow arrow, the point where the greater occipital nerve exits. Red arrow, the point where the occipital artery exits. The black dotted line shows the boundaries of the muscles.

*Subtype 1‐D*: GON pierces fibers of each TM and SS at one point, no cross of GON and OA exists (Figure 4).

**FIGURE 5:**
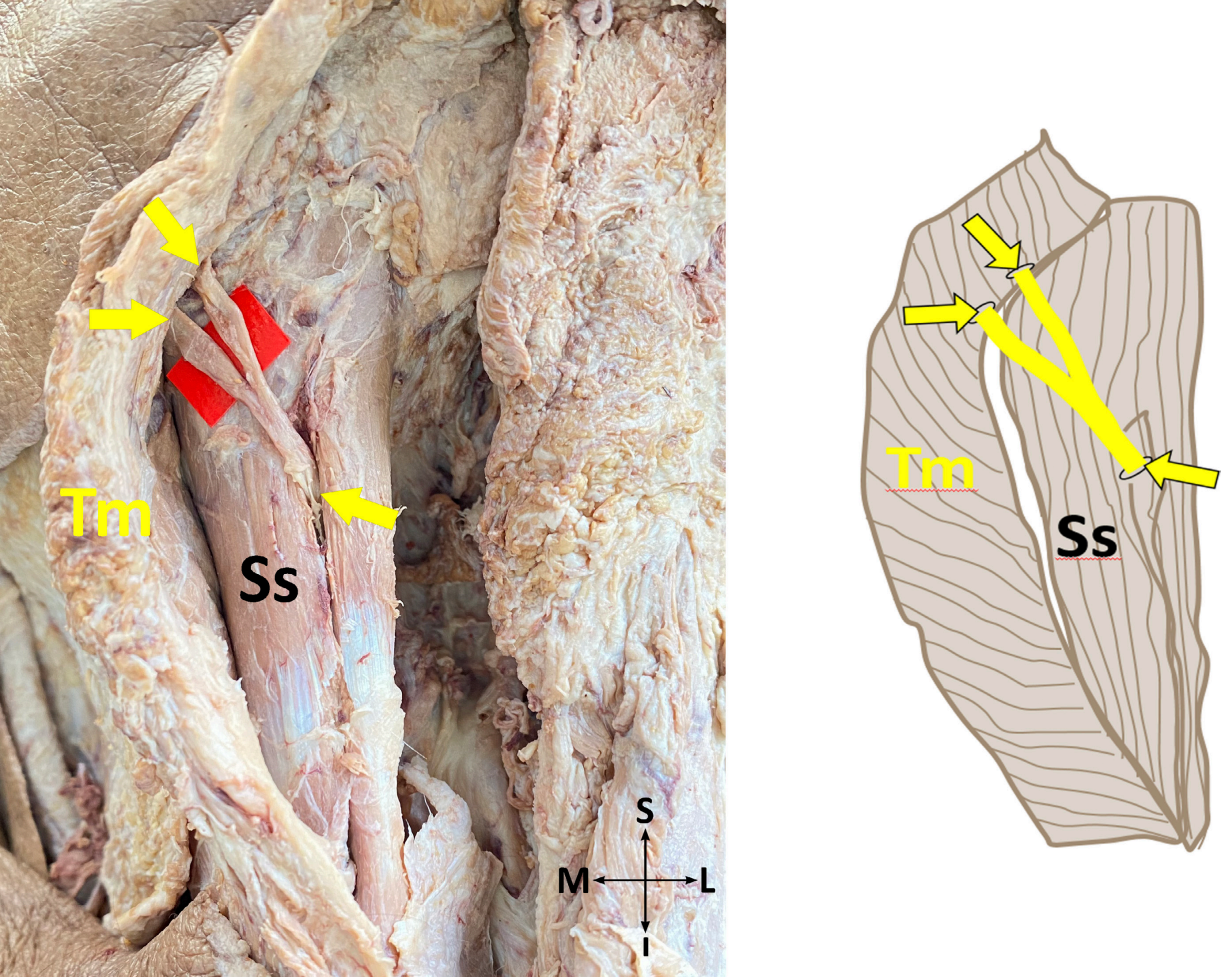
Trapezius muscle is pierced by the greater occipital nerve at two different points on the cadaver (on the left side), and its schematic illustration (on the right side) (posterior view, left). I, inferior; L, lateral; M, medial; S, superior; SS, semispinalis capitis muscle; TM, trapezius muscle; Yellow arrows, the points where the greater occipital nerve exits from the semispinalis capitis muscle, and the superior and inferior branches of the nerve enter the fibers of the trapezius muscle.

*Subtype 1‐E*: GON pierces fibers of TM at two different points (Figure 5) and it pierces fibers of the SS at one point, a single cross of GON and OA exists.

**FIGURE 6:**
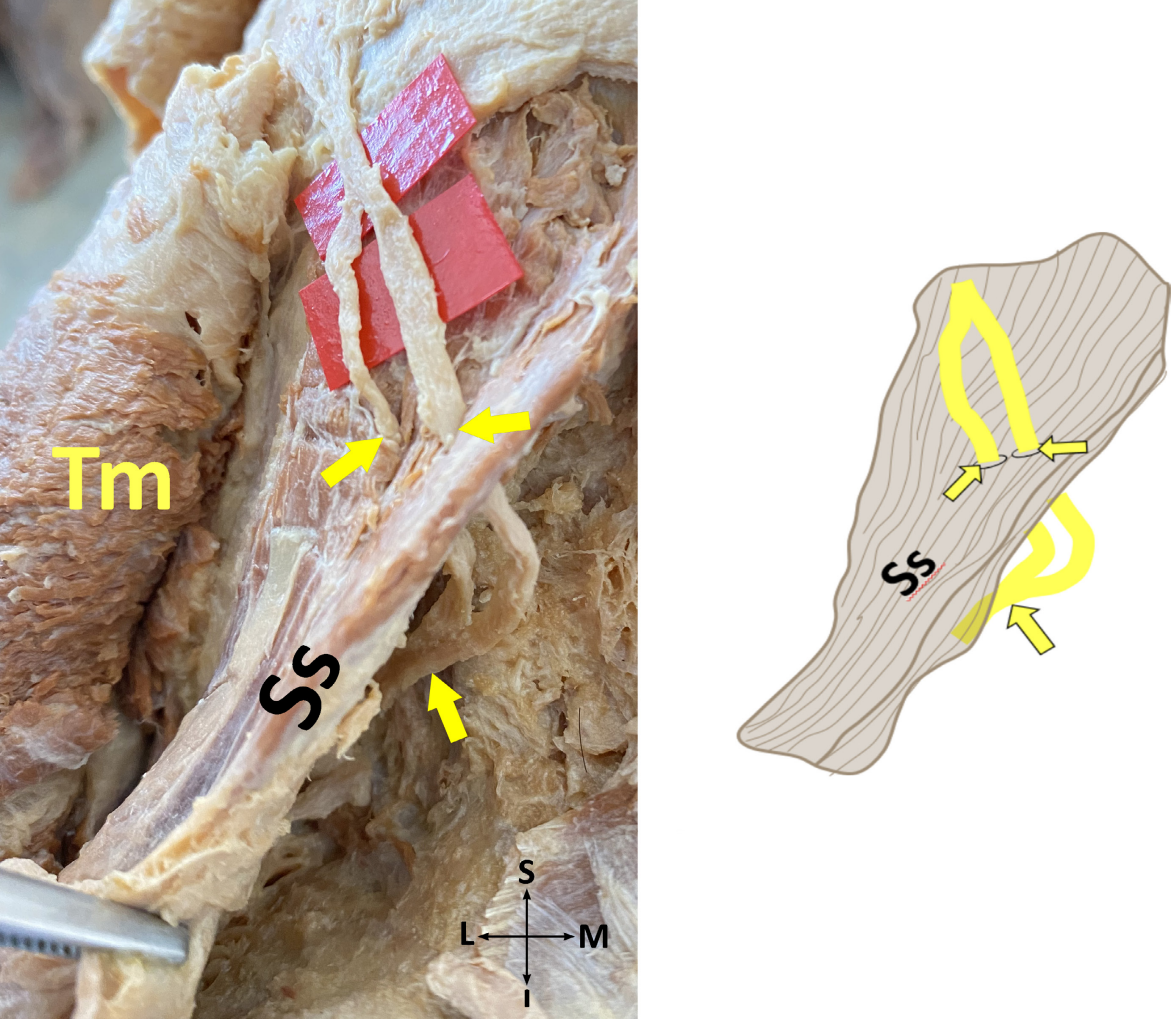
Semispinalis capitis muscle is pierced by the greater occipital nerve at two different points on the cadaver (on the left side), and its schematic illustration (on the right side) (posterior view, left). I, inferior; L, lateral; M, medial; S, superior; SS, semispinalis capitis muscle; TM, trapezius muscle; Yellow arrows, greater occipital nerve, and the exit points of the superior and inferior branches of the greater occipital nerve from the semispinalis capitis muscle.

*Subtype 1‐F*: GON pierces fibers of TM at one point and it pierces fibers of SS at two different points (Figure 6), no cross of GON and OA exists.

*Type 2*: GON pierces aponeurosis of TM, curves around the lower edge of OCI and it is attached loosely to this muscle.

*Subtype 2‐A*: GON pierces aponeurosis of each TM and fibers of SS at one point, a single cross of GON and OA exists (Figure 1).

*Subtype 2‐B*: GON pierces aponeurosis of each TM and fibers of SS at one point, two crosses of GON and OA exist (Figure 2).

*Subtype 2‐C*: GON pierces aponeurosis of each TM and fibers of SS at one point, three crosses of GON and OA exist (Figure 3).

*Subtype 2‐D*: GON pierces aponeurosis of each TM and fibers of SS at one point, no cross of GON and OA exists (Figure 4).

**FIGURE 7:**
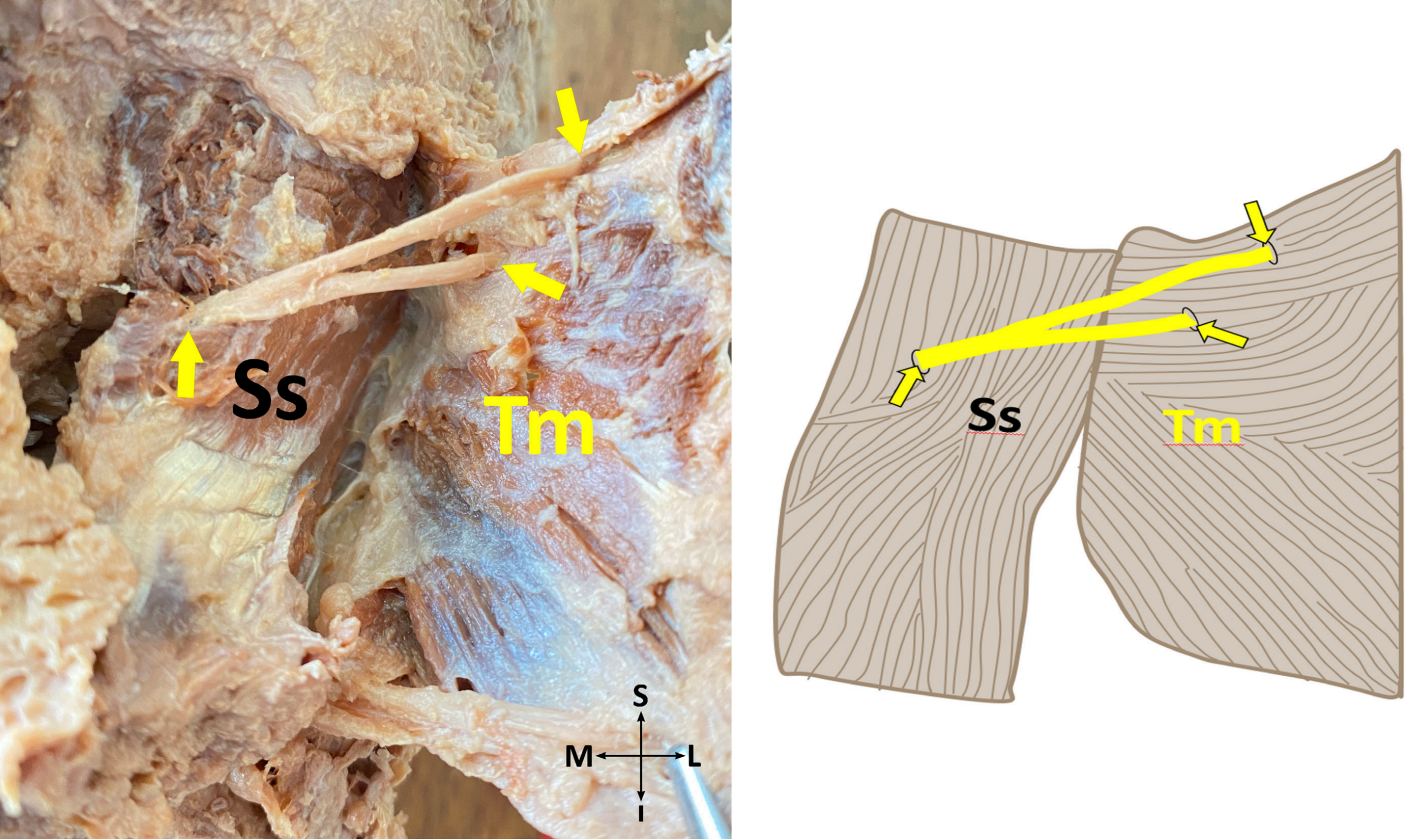
Aponeurosis of the trapezius muscle is pierced by the greater occipital nerve at two different points on the cadaver (on the left side), and its schematic illustration (on the right side) (posterior view, right). I, inferior; L, lateral; M, medial; S, superior; SS, semispinalis capitis muscle; TM, trapezius muscle; Yellow arrows, the points where the greater occipital nerve exits from the semispinalis capitis muscle, and the superior and inferior branches of the nerve enter the aponeurosis of the trapezius muscle.

*Subtype 2‐E*: GON pierces aponeurosis of TM at two different points (Figure 7) and fibers of SS at one point, a single cross of GON and OA exists.

*Subtype 2‐F*: GON pierces aponeurosis of TM at one point and fibers of SS at two different points (Figure 6), a single cross of GON and OA exists.

*Type 3*: GON pierces the aponeurosis located between TM and SCM at one point, curves around the lower edge of OCI and it is attached loosely to OCI, perforates fibers of each TM and SS at one point, a single cross of GON and OA exists.

*Type 4*: GON curves around the lower edge of OCI and is connected very tightly to this muscle.

*Subtype 4‐A*: GON pierces aponeurosis of each TM and fibers of SS at one point, a single cross of GON and OA exists (Figure 1).

*Subtype 4‐B*: GON pierces aponeurosis of each TM and fibers of SS at one point, two crosses of GON and OA exist (Figure 2).

*Subtype 4‐C*: GON pierces aponeurosis of each TM and fibers of SS at one point, three crosses of GON and OA exist (Figure 3).

*Subtype 4‐D*: GON pierces aponeurosis of each TM and fibers of SS at one point, no cross of GON and OA exists (Figure 4).

*Subtype 4‐E*: GON pierces aponeurosis of TM at two different points (Figure 7) and fibers of SS at one point, a single cross of GON and OA exists.

*Subtype 4‐F*: GON perforates aponeurosis of TM at one point and fibers of SS at two different points (Figure 6), a single cross of GON and OA exists.

After identifying a classification, distribution of numbers and frequency of main types and subtypes of GON in terms of gender and side was determined.

In addition, distribution of number and frequency of number of cross(es) of GON and OA in terms of gender and side was investigated.

Moreover, considering the transverse line that passes from the EOP, the location of the cross(es) of GON and OA (either above or below this line) and the location of the emerging point of GON from TM (either above or below this line) were identified and noted.

Furthermore, if GON and OA crossed each other, their relationship with each other just before their first cross was investigated. If they did not cross each other, their relationship with each other during their course was noted.

Lastly, the number of branches of GON up to SS, the number of branches of GON that were sent to SS, and the number of branches of GON that were sent to TM were determined.

### 2.2 Statistical evaluation

For evaluation and analysis of data, IBM Statistical Package for the Social Sciences (SPSS) Statistics 21.0 was used. Descriptive statistics of categorical variables were revealed with frequency and percent, and continuous variables were expressed with mean, standard deviation, or median (minimum and maximum). The Chi‐square test was used to analyze categorical variables in terms of types (Types 1, 2, and 4). Since there was one side in the Type 3 morphology group, the Type 3 variable was not included in the analysis. Similarly, the gender and side comparisons of the number of cross(es) of the GON and OA were made using the Chi‐square test by reducing the number of cross(es) to two groups (no cross and cross). In the case of presence of more than two groups (no cross, single cross, two crosses…), statistical significance could not be analyzed. Moreover, the Chi‐square test was again used to analyze the position of the cross(es) of the GON and OA in relation to (above or below) the transverse line from the EOP in terms of gender and side. The statistical significance level was accepted as *p* ≤ 0.05.

## 3 RESULTS

Distribution of numbers and frequency of main types of GON in terms of gender and side is tabulated in Table 1 and the similar distribution belonging to subtypes of types 1, 2, and 4 are given in Tables 2, 3, 4, respectively. No statistically significant difference was observed between the sides in terms of types (*p* = 0.717). Similarly, no statistically significant difference was found between the genders in terms of types (*p* = 0.141).

**TABLE 1:** Distribution of numbers and frequency of main types of the greater occipital nerve in terms of gender and side.

	*n* (side)	Type 1	Type 2	Type 3	Type 4
Male	54	9 (16.7%)	41 (76%)	1 (1.8%)	3 (5.5%)
Female	28	2 (7.1%)	20 (71.4%)	—	6 (21.5%)
Right	41	6 (14.7%)	29 (70.7%)	1 (2.4%)	5 (12.2%)
Left	41	5 (12.2%)	32 (78%)	—	4 (9.8%)
Total	82	11 (13.4%)	61 (74.4%)	1 (1.2%)	9 (11%)

*Note*: *Type 1*: Greater occipital nerve pierces fibers of trapezius muscle, curves around lower edge of obliquus capitis inferior muscle, and it is attached loosely to this muscle. *Type 2*: Greater occipital nerve pierces aponeurosis of trapezius muscle, curves around the lower edge of obliquus capitis inferior muscle, and it is attached loosely to this muscle. *Type 3*: Greater occipital nerve pierces the aponeurosis located between trapezius and sternocleidomastoid muscles at one point, curves around the lower edge of obliquus capitis inferior muscle, and it is attached loosely to obliquus capitis inferior muscle, perforates fibers of each trapezius and semispinalis capitis muscles at one point, a single cross of greater occipital nerve and occipital artery exists. *Type 4*: Greater occipital nerve curves around the lower edge of obliquus capitis inferior muscle and is connected very tightly to this muscle.

**TABLE 2:** Distribution of numbers and frequency of subtypes of Type 1 of the greater occipital nerve in terms of gender and side.

	*n* (side)	Subtype 1‐A	Subtype 1‐B	Subtype 1‐C	Subtype 1‐D	Subtype 1‐E	Subtype 1‐F
Male	9	5 (55.6%)	2 (22.2%)	1 (11.1%)	—	1 (11.1%)	—
Female	2	1 (50%)	—	—	—	—	1 (50%)
Right	6	5 (83.3%)	1 (16.7%)	—	—	—	—
Left	5	1 (20%)	1 (20%)	1 (20%)	—	1 (20%)	1 (20%)
Total	11	6 (54.5%)	2 (18.2%)	1 (9.1%)	—	1 (9.1%)	1 (9.1%)

*Note*: *Type 1*: Greater occipital nerve pierces fibers of trapezius muscle, curves around lower edge of obliquus capitis inferior muscle, and it is attached loosely to this muscle. *Subtype 1‐A*: Greater occipital nerve pierces fibers of each trapezius muscle and semispinalis capitis muscle at one point, a single cross of greater occipital nerve and occipital artery exists. *Subtype 1‐B*: Greater occipital nerve pierces fibers of each trapezius muscle and semispinalis capitis muscle at one point, two crosses of greater occipital nerve and occipital artery exist. *Subtype 1‐C*: Greater occipital nerve pierces fibers of each trapezius muscle and semispinalis capitis muscle at one point, three crosses of greater occipital nerve and occipital artery exist. *Subtype 1‐D*: Greater occipital nerve pierces fibers of each trapezius muscle and semispinalis capitis muscle at one point, no cross of greater occipital nerve and occipital artery exists. *Subtype 1‐E*: Greater occipital nerve pierces fibers of trapezius muscle at two different points and it pierces fibers of the semispinalis capitis muscle at one point, a single cross of greater occipital nerve and occipital artery exists. *Subtype 1‐F*: Greater occipital nerve pierces fibers of trapezius muscle at one point and it pierces fibers of semispinalis capitis muscle at two different points, no cross of greater occipital nerve and occipital artery exists.

**TABLE 3:** Distribution of number and frequency of subtypes of Type 2 of the greater occipital nerve in terms of gender and side.

	*n* (side)	Subtype 2‐A	Subtype 2‐B	Subtype 2‐C	Subtype 2‐D	Subtype 2‐E	Subtype 2‐F
Male	41	34 (83%)	3 (7.4%)	1 (2.4%)	2 (4.8%)	—	1 (2.4%)
Female	20	10 (50%)	—	1 (5%)	5 (25%)	1 (5%)	3 (15%)
Right	29	20 (69%)	1 (3.4%)	1 (3.4%)	4 (13.9%)	1 (3.4%)	2 (6.9%)
Left	32	24 (75%)	2 (6.3%)	1 (3.1%)	3 (9.3%)	—	2 (6.3%)
Total	61	44 (72.1%)	3 (4.9%)	2 (3.4%)	7 (11.5%)	1 (1.6%)	4 (6.5%)

*Note*: *Type 2*: Greater occipital nerve pierces aponeurosis of trapezius muscle, curves around the lower edge of obliquus capitis inferior muscle, and it is attached loosely to this muscle. *Subtype 2‐A*: Greater occipital nerve pierces aponeurosis of each trapezius muscle and fibers of semispinalis capitis muscle at one point, a single cross of greater occipital nerve and occipital artery exists. *Subtype 2‐B*: Greater occipital nerve pierces aponeurosis of each trapezius muscle and fibers of semispinalis capitis muscle at one point, two crosses of greater occipital nerve and occipital artery exist. *Subtype 2‐C*: Greater occipital nerve pierces aponeurosis of each trapezius muscle and fibers of semispinalis capitis muscle at one point, three crosses of greater occipital nerve and occipital artery exist. *Subtype 2‐D*: Greater occipital nerve pierces aponeurosis of each trapezius muscle and fibers of semispinalis capitis muscle at one point, no cross of greater occipital nerve and occipital artery exists. *Subtype 2‐E*: Greater occipital nerve pierces aponeurosis of trapezius muscle at two different points and fibers of semispinalis capitis muscle at one point, a single cross of greater occipital nerve and occipital artery exists. *Subtype 2‐F*: Greater occipital nerve pierces aponeurosis of trapezius muscle at one point and fibers of semispinalis capitis muscle at two different points, a single cross of greater occipital nerve and occipital artery exists.

**TABLE 4:** Distribution of number and frequency of subtypes of Type 4 of the greater occipital nerve in terms of gender and side.

	*n* (side)	Subtype 4‐A	Subtype 4‐B	Subtype 4‐C	Subtype 4‐D	Subtype 4‐E	Subtype 4‐F
Male	3	1 (33.3%)	1 (33.3%)	—	—	1 (33.4%)	—
Female	6	4 (66.6%)	—	—	1 (16.7%)	—	1 (16.7%)
Right	5	2 (40%)	1 (20%)	—	1 (20%)	1 (20%)	—
Left	4	3 (75%)	—	—	—	—	1 (25%)
Total	9	5 (55.6%)	1 (11.1%)	—	1 (11.1%)	1 (11.1%)	1 (11.1%)

*Note*: *Type 4*: Greater occipital nerve curves around the lower edge of obliquus capitis inferior muscle and is connected very tightly to this muscle. *Subtype 4‐A*: Greater occipital nerve pierces aponeurosis of each trapezius muscle and fibers of semispinalis capitis muscle at one point, a single cross of greater occipital nerve and occipital artery exists. *Subtype 4‐B*: Greater occipital nerve pierces aponeurosis of each trapezius muscle and fibers of semispinalis capitis muscle at one point, two crosses of greater occipital nerve and occipital artery exist. *Subtype 4‐C*: Greater occipital nerve pierces aponeurosis of each trapezius muscle and fibers of semispinalis capitis muscle at one point, three crosses of greater occipital nerve and occipital artery exist. *Subtype 4‐D*: Greater occipital nerve pierces aponeurosis of each trapezius muscle and fibers of semispinalis capitis muscle at one point, no cross of greater occipital nerve and occipital artery exists. *Subtype 4‐E*: Greater occipital nerve pierces aponeurosis of trapezius muscle at two different points and fibers of semispinalis capitis muscle at one point, a single cross of greater occipital nerve and occipital artery exists. *Subtype 4‐F*: Greater occipital nerve perforates aponeurosis of trapezius muscle at one point and fibers of semispinalis capitis muscle at two different points, a single cross of greater occipital nerve and occipital artery exists.

No aneurysm or structural abnormality was observed in OA. Distribution of number and frequency of number of cross(es) of GON and OA in terms of gender and side is stated in Table 5. The crossing of the GON and OA (no cross and cross) was statistically significant in terms of gender (*p* = 0.011), but not statistically significant in terms of side (*p* = 0.724).

**TABLE 5:** Distribution of number and frequency of number of cross(es) of the greater occipital nerve and occipital artery in terms of gender and side.

	*n* (side)	No cross	Single cross	Two cross	Three cross
Male	54	2 (3.7%)	44 (81.5%)	6 (%11.1)	2 (%3.7)
Female	28	7 (25%)	20 (71.4%)	—	1 (%3.6)
Right	41	5 (12.1%)	32 (78%)	3 (%7.4)	1 (%2.5)
Left	41	4 (9.7%)	32 (78%)	3 (%7.4)	2 (%4.9)
Total	82	9 (10.9%)	64 (78%)	6 (%7.4)	3 (%3.7)

Considering the transverse line that passes from the EOP, distribution of number and frequency of the location of the cross(es) of GON and OA (either above or below this line) in terms of gender and side are given in Table 6. Considering the transverse line that passed from the EOP, the location of the emerging point of GON from TM (either above or below this line) was determined to be superior to this line on only one side (left, 1.2%). In the rest of the cases (81 sides; 41 right and 40 left, 98.8%), this emerging point was determined to be inferior to this line. The position of the cross(es) of the GON and OA in relation to (above or below) the transverse line passing from the EOP was not statistically significant in terms of gender (*p* = 0.329) and side (*p* = 0.515).

**TABLE 6:** Considering the transverse line that passes from the external occipital protuberance, distribution of number and frequency of the location of the cross(es) of the greater occipital nerve and occipital artery (either above or below this line) in terms of gender and side.

	*n* (number of cross)	Above	Below
Male	62	14 (%22.6)	48 (%77.4)
Female	23	3 (%13.1)	20 (%86.9)
Right	41	7 (%17)	34 (%83)
Left	44	10 (%22.7)	34 (%77.3)
Total	85	17 (%20.7)	68 (%79.3)

Related with the relationship of GON and OA, it was observed that GON coursed laterally to the OA on 11 sides (three right and eight left sides; 13.4%) of the cases, and GON was medial to OA on 71 sides (38 right and 33 left sides; 86.6%).

GON gave at least one, maximum of four branches on 69 sides up to the SS muscle. GON sent at least one and at most three branches to the SS muscle in 67 sides (median = 1). On 32 sides, GON sent at least one and at most two branches to TM in 32 sides (median = 1).

## 4 DISCUSSION

### 4.1 Types of GON

**FIGURE 8:**
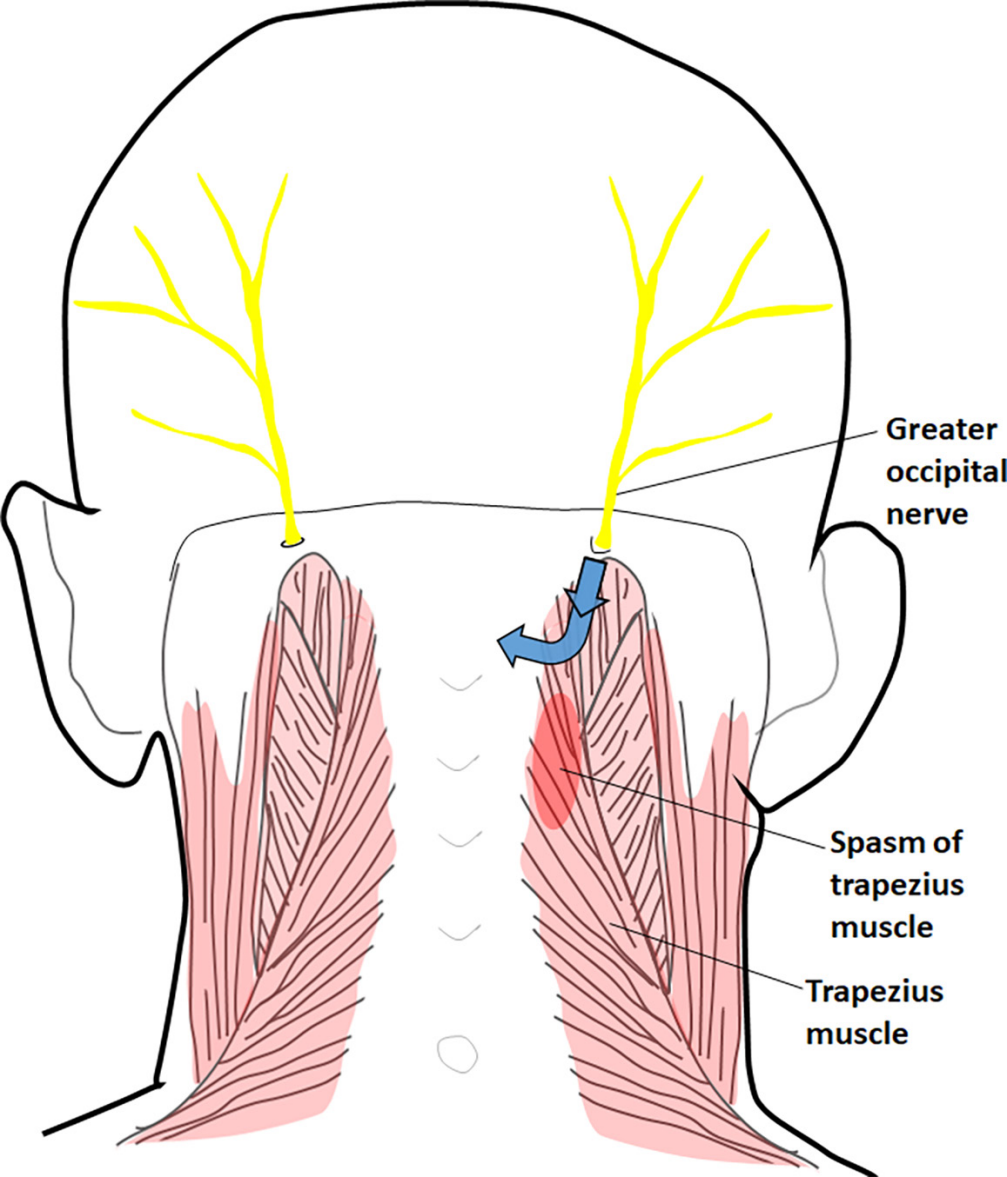
Demonstration of trapezius muscle spasm pulling the aponeurotic sling between the trapezius and sternocleidomastoid muscles down and away from the greater occipital nerve and thus not squeezing this nerve (posterior view). The blue arrows indicate the direction of traction of the spasm.

Research related with the morphological classification of GON is quite limited and a standard classification has not been accepted by the researchers yet. Bogduk (1981) reported that a single type of GON was identified in his study. He highlighted that none of the GONs pierced TM on five adult cadavers he had dissected and he added that GONs had emerged from the opening at the upper border of the aponeurotic sling extending between SCM and TM. In their study which was performed on nine adult cadavers (18 sides) fixed with formalin, Vital et al. (1989) reported that all GONs passed through the tendinous part of TM and they confirmed the results of Bogduk (1981), identifying a single type. On the other hand, in their autopsy study of 20 adult cadavers (40 sides), Bovim et al. (1991) determined that GON pierced TM on 18 (45%) sides and did not pierce TM on 22 (55%) sides, thus they identified two different types of GON. Concerning the relationship of GON with TM, for the first time, Tubbs et al. (2014) emphasized the notion of types and subtypes of GON, and reported that GON pierced TM on five (16.7%) sides (identified by them as Type 1) and traveled through the aponeurosis of TM on 25 (83.3%) sides (identified by them as Type 2). Won et al. (2018) did not use the concept of “type” that was pointed out by Tubbs et al. (2014). Instead, in their cadaveric study, Won et al. (2018) recorded that GON penetrated TM on 14 (25%) sides, it traveled through the aponeurosis of TM on seven (12.5%) sides, and it ran to the lateral border of TM and pierced the fascia extending between the cranial part of this muscle and SCM on 35 (62.5%) sides. After the study of Tubbs et al. (2014), the concept of “type” related with the relationship of GON and TM was also remarkable in the study of Huanmanop et al. (2021). They revealed that the aponeurosis of TM, TM itself, and the aponeurosis extending between SCM and TM were pierced by GON at 67 (67%) sides, 2 (2%) sides, and 31 (31%) sides, respectively. Without making a type classification, Lainé et al. (2022) reported that GON passed through TM on six sides (75%) and the aponeurosis of TM on two sides (25%). In the present study, GON pierced the aponeurosis of TM (*Type 2*: the most common variant was that the GON pierced the aponeurosis of TM, curved around the lower edge of the OCI, and was loosely attached to the OCI; and *Type 4*: GON pierced aponeurosis of each TM and fibers of SS at one point, a single cross of GON and OA existed) on 70 (85.4%) sides, TM itself (*Type 1*: GON pierced fibers of TM, curved around lower edge of OCI, and it was attached loosely to this muscle) on 11 (13.4%) sides, and the aponeurosis extending between SCM and TM (*Type 3*: GON pierced the aponeurosis located between TM and SCM at one point, curved around the lower edge of OCI and it was attached loosely to OCI, perforated fibers of each TM and SS at one point, a single cross of GON and OA existed) on one (1.2%) side. Our results are compatible with the findings of Tubbs et al. (2014). According to Bogduk (1981), since GONs did not pierce TM and they arose from the aponeurotic sling extending between SCM and TM, a spasm of TM would pull this aponeurotic sling downward and away from GON, so that it would not impinge GONs (Figure 8). Due to the fact that Type 3 GON of our study, in which GON pierced the aponeurosis which was located between TM and SCM at one point, had been determined at a quite low frequency (1.2%), we considered that the possibility noted by Bogduk (1981) was rare. Moreover, Type 1 GON of our study in which GON pierced the fibers of TM had been determined at a significant rate (13.4%). Due to a spasm of type 1 GON, TM might squeeze GONs and cause cervicogenic headaches. Vital et al. (1989) emphasized that in case of tendinitis of TM, entrapment neuropathy of GON would develop in GONS piercing the aponeurosis of TM. As we have determined that the frequency of GONs piercing the aponeurosis of TM is 85.4% (the highest frequency of our GONs), we support that the frequency of entrapment neuropathy of GON at this point may be high, as it has been already highlighted by Vital et al. (1989).

In our study, no statistically significant difference was obtained between types and sides and between types and genders (*p* > 0.05). This may reflect the similar embryological development of GON in terms of gender and side and could mean that the phenotypic sex and side of the cadaver had no impact on the anatomical scheme that we observed. In other words, according to our results, the clinical applications for GON may not significantly differ in terms of gender and side.

### 4.2 Relationship between GON and OCI

Regarding the relationship between OCI and GON, in their autopsy studies, Bovim et al. (1991) recorded that GON ascended by curving from the lower border of OCI on 37 (92.5%) sides, and pierced OCI near the lower edge of this muscle on three (7.5%) sides. Natsis et al. (2006) determined that GON rose by turning from the lower border of OCI on 76 sides (95%) and pierced OCI on three (3.75%) sides, and passed through the suboccipital triangle on one (1.25%) side. Tubbs et al. (2014) reported that GON ascended by curving from the lower border of OCI on 28 (93.3%) sides, and pierced OCI near the lower edge of this muscle on two (6.7%) sides. Huanmanop et al. (2021) stated that GON rose by curving from the lower border of OCI on 94 (94%) sides, and pierced OCI near the lower edge of this muscle on six (6%) sides. Scherer et al. (2019) determined that GON ascended by curving from the lower border of OCI on 38 (95%) sides and pierced OCI on two (5%) sides. In addition, unlike previous studies, GONs ascending by turning from the lower edge of OCI were subdivided into two separate subgroups by Scherer et al. (2019); they reported that GON was loosely connected to OCI on seven (17.5%) sides and firmly attached either to the muscle itself or to its fascia on 31 (77.5%) sides. Lainé et al. (2022) noted that GON rose by turning from the lower margin of OCI in all four (8 sides) cases (100%). In our study, the relationship between OCI and GON was examined, and the fact that GON was attached too tightly (Type 4) or loosely to OCI (other main types) was investigated, and the results were determined on 9 (10.9%) sides and 73 (89.1%) sides, respectively. We think that the morphological characteristics of the GONs penetrating the OCI reported by the researchers correspond to GONs which are very tightly attached to OCI in our samples. Consequently, we think that our results are compatible with the results of Bovim et al. (1991). In the present study, although GON attaching to OCI very tightly has a low frequency (10.9%), it may reveal that OCI may act as a pivotal point for GON (Cesmebasi et al., 2015; Janis et al., 2010; Lambru et al., 2014; Mosser et al., 2004). In falls, blunt traumas, and whiplash injuries, this pivotal point may also play an important role in the pathogenesis of occipital neuralgia (Magnússon et al., 1996). Moreover, the tight attachment point of GON to OCI may be a potential compression point for this nerve. Actually, Janis et al. (2010) reported this point to be the first proximal potential entrapment point of GON. In this context, if there is no response to GONB, the possibility of GON to be compressed at the returning point from the lower edge of OCI should be considered.

### 4.3 Relationship between GON and SS and TM

In their study on 112 patients and 13 cadavers (125 cases and 250 sides), Ducic et al. (2009) recorded that GON pierced SS on two points by two branches of it, on 15 (6.1%) sides and then these two branches often reconnected and/or GON entered the “trapezial tunnel” in TM, as two branches it gave just before entering the tunnel. Bovim et al. (1991) noted that some of the GONs branched off before reaching TM. Natsis et al. (2006) observed that at two sides (at two half heads), GON split into two branches and pierced TM at two different points, and then merged. In our study, GON pierced SS at two different points on six (7.3%) sides and penetrated TM or its aponeurosis in two separate points on three (3.6) sides. Our results highly correlate with the previous results. Bovim et al. (1991) declared that if SS and TM were pierced by GON in two different points, they might allow compression of these distinct branches. We believe that such variations may complicate the block and decompression surgery of GON. In decompression surgery, missing any branch may reduce the effectiveness of the practice. At interventions where GON is “liberalized”, such variations may be a reason for some patients remaining refractory.

### 4.4 Relationship between GON and OA

Lainé et al. (2022) noted that GON and OA crossed each other on six sides (75%), while the nerve and artery were parallel to each other without crossing on two sides (25%). In our study, one cross, two crosses, and three crosses of GON and OA were determined on 64 (78%), 6 (7.4%), and 3 sides (3.7%), respectively, and the cross of them was absent on nine (10.9%) sides (Table 5). When our results are compared with the ones of Lainé et al. (2022), frequency of crosses of our study is higher. This fact may probably be caused by the differences in sample size and/or race. We believe that in migraine, occipital neuralgia, and cervicogenic headache if there is not any cross of GON and OA, the compression point is likely to be located more proximally. In such cases, focusing on the proximal part of GON rather than the distal part may provide success in decompression procedures. We found that crossing of GON and OA (cross versus no cross) was statistically significant between genders (*p* < 0.05) and not statistically significant between the sides (*p* > 0.05). The fact that crossing of GON and OA (cross versus no cross) was statistically significant between the sexes may indicate that the process of crossing of the nerve and the artery during embryological development might be different between the sexes. In other words, our result is that crossing of GON and OA has been statistically more common in males than in females. This result does not correspond to the fact that headache is more common in females than in males worldwide. We think that this may be due to the fact that crossing of GON and OA has little effect among the causes of headaches. In addition, according to the results of our study, we believe that in clinical applications for the distal part of the GON, it may be useful to keep in mind that males more frequently have crossing of GON and OA than females and interventional planning should be made accordingly. When crossing of GON and OA is considered from sides point of view, it can be said that related embryological process does not create any developmental differences between the sides.

GONB can be used for both diagnosis and treatment (Martelletti & van Suijlekom, 2004; Scherer et al., 2019; Young, 2010), and the success of it requires an accurate determination of the injection site (Won et al., 2018). In the “blind technique”, the injection site is described to be the midpoint where GON crosses superior nuchal line or as the point between 1/3 medial and 1/3 middle of the line extending between EOP and mastoid process (Austad, 2004; Won et al., 2018; Young, 2010). In order to determine the place for application of GONB, the topographic location of cross of GON and OA may be used as a certain landmark. It was reported in previous studies that EOP, which we used to predict the location of cross of GON and OA, was one of the certain landmarks for deciding where interventions for GON might be applied (Austad, 2004; Caputi & Firetto, 1997; Mosser et al., 2004) and GON related procedures were usually applied caudal to EOP. Nevertheless, our results reveal that the cross of GON and OA can be located at different points. Although most of the crosses of GON and OA (68 sides, 79.3%) are located below the transverse line passing from EOP, a significant number of them (17 sides, 20.7%) are located above the transverse line, thus we believe that this fact might cause GONB to be inconclusive and a new treatment plan should be made accordingly. Moreover, in our study, on one side (1.2%), we observed that emerging of GON at TM was located above the transverse line passing from EOP. In the remaining 81 sides (98.8%), these emerging points were located below this line. Since the exit of GON from TM is the point where the nerve becomes superficial, it has been the main target of clinical applications (Dach et al., 2015; Weibelt et al., 2010). Although this result has been achieved at a low rate (1.2%), it may affect the outcome of clinical applications. In our study, the position of the cross(es) of the GON and OA in relation to (above versus below) the transverse line passing from the EOP was not statistically significant in terms of gender (*p* > 0.05) and side (*p* > 0.05). This situation might be because of similar embryological development in genders and sides. Consequently, we believe that clinical applications related with the topographic location of this crossing may not be affected by gender or side significantly.

As the OA can be quickly and easily found with the help of fingers without any device in an inexpensive way, this artery itself has also been defined to be a reliable landmark at GONB (Murphy, 1988; Vanterpool et al., 2020; Young, 2010). However, the pulse of OA occasionally cannot be taken with the help of a finger (Arai et al., 2013). Although previous studies have focused on the relationship between GON and OA (Chung et al., 2022; Lainé et al., 2022; Shimizu et al., 2007; Won et al., 2018), a few research (Lainé et al., 2022; Won et al., 2018) have mentioned the relationship of them either being located medial or lateral to each other, reporting that the OA is typically located lateral to GON (Nair et al., 2018; Won et al., 2018). Nevertheless, Won et al. (2018) reported that OA was medial to GON in 14.3% of the cases (56 sides). Similarly, Lainé et al. (2022) reported that OA was medial to GON in 50% of the cases (four sides), emphasizing a higher frequency of this situation. Neither Won et al. (2018) nor Lainé et al. (2022) did not specify whether this relationship was before or after the cross of GON and OA. The results of our study, reporting that GON was lateral to OA on 11 (13.4%) sides, whereas it was medial to OA on 71 (86.6%) sides, confirm the results of Won et al. (2018). Vanterpool et al. (2020) applied the “modified blind technique” to 50 patients and stated that approximately 20% of cases did not respond to treatment. This rate could be evaluated close to the rate of approximately 15% in which GON was located lateral to the OA, suggesting that such variation would cause failure to respond to treatment.

### 4.5 Branches of GON

Junewicz et al. (2013) reported intramuscular or premuscular branching of GON in 7.4% (20 patients) of 272 migraine patients. Kastler et al. (2018) examined MR images of 20 healthy subjects to detect GON and found that only four cases had motor branches of that nerve. They highlighted that these might be anatomical variations of the motor branches of GON. In our study, branches of GON were encountered quite frequently (median = 1), which was up to four branches in some cases. In the literature, we could not find any papers to compare with our results. Junewicz et al. (2013) noted that they observed the branching pattern in their study only at migraine patients and that this might play a role in the pathogenesis of migraine. Since we do not have any information on the medical history of our cadavers concerning migraine, we cannot make any comments on this issue. Each additional branch of GON can be a potential entrapment point for this nerve, and the risk of migraine may increase in such cases (Junewicz et al., 2013). As the number of branches we have observed in our study has been often high, a wide territory may be at risk. Additional GONB for this broad field may be required. Similarly, these branches may need to be considered in decompression surgery. One of the reasons why interventions have limited success may be due to these branches. We have no idea whether the branches of GON that we have observed include motor fibers as we have not searched. Further studies are needed for this aim.

#### 4.5.1 Limitation(s) of this study

We have not had any data regarding the clinical presentation of our cadavers. If we have had, it would be interesting to see if a particular type or subtype having a particular clinical presentation.

## 5 CONCLUSION

In our study, unlike previous studies, the types and subtypes of GON were identified in a detailed way and they were analyzed in terms of gender and side. Moreover, the possible clinical implications of the morphological features of GON were asserted. The relationship between GON and OCI was discussed in detail as well as the relationship between GON and SS, TM, and aponeurosis of the trapezius and the aponeurosis extending between the SCM and TM. The relationship between GON and OA was also examined. In this context, the position and number of the cross(es) of GON and OA, and the topographic location of the cross(es) in relation to the line passing from the EOP were detected. Furthermore, the position and number of the cross(es) of GON and OA were analyzed in terms of gender and side. The position (medial–lateral) of the GON and OA (relative to each other) was determined. The branches of the GON were investigated. The number of branches of the GON up to SS was noted and the number of branches of GON that were sent to SS and the number of branches of GON that were sent to TM were determined.

As a conclusion, this study documents the largest report of detailed morphological classification of GON. Although our study confirms many previous anatomical findings, it also explores anatomical properties of GON that have not yet been studied and indicates several new anatomical findings.

## AUTHOR CONTRIBUTIONS

Latif Saglam and Ozcan Gayretli contributed to the conceptual design of the study, network analysis, data interpretation, and manuscript preparation. Osman Coskun contributed to the acquisition of data and manuscript preparation. Aysin Kale contributed to the data analysis/interpretation and critical revision of the manuscript.

## Data Availability

We have shared new data alongside their paper.
